# Corneal Biomechanical Assessment Using Corneal Visualization Scheimpflug Technology in Keratoconic and Normal Eyes

**DOI:** 10.1155/2014/147516

**Published:** 2014-03-30

**Authors:** Lei Tian, Yi-Fei Huang, Li-Qiang Wang, Hua Bai, Qun Wang, Jing-Jing Jiang, Ying Wu, Min Gao

**Affiliations:** Department of Ophthalmology, Chinese PLA General Hospital, Fuxing Road 28, Beijing 100853, China

## Abstract

*Purpose*. To compare the corneal biomechanical properties of keratoconic patients and age-matched controls using corneal visualization Scheimpflug technology (Corvis ST). *Methods*. Sixty keratoconic eyes from 47 keratoconus patients and 60 normal eyes from 60 controls were enrolled in this prospective study. Tomography and biomechanical parameters of all eyes were obtained with the Pentacam and Corvis ST, respectively. Intraocular pressure was measured using a Goldmann applanation tonometer. *Results.* The tomography and biomechanical parameters of the keratoconic corneas were significantly different from those of the normal corneas except for the anterior chamber angle, first applanation length, the highest concavity time, and peak distance. The deformation amplitude was the best predictive parameter (area under the curve: 0.882), with a sensitivity of 81.7%, although there was a significant overlap between keratoconic and normal corneas that ranged from 1.0 to 1.4 mm. In both the keratoconus and control groups, the deformation amplitude was negatively correlated with intraocular pressure, central corneal thickness, and corneal volume at 3 and 5 mm. *Conclusions*. Corvis ST offers an alternative method for measuring corneal biomechanical properties. The possibility of classifying keratoconus based on deformation amplitude deserves clinical attention.

## 1. Introduction

Keratoconus is an ectatic corneal disorder characterized by a progressive noninflammatory thinning of the corneal stroma, which results in corneal protrusion, irregular astigmatism, and decreased vision [1]. It is usually bilateral but asymmetrical with typical onset at puberty within a given population [2, 3]. The loss or slippage of collagen fibrils and interfibrillary substance in the corneal stroma of keratoconus patients can lead to biomechanical instability of the cornea with consequent changes in the cornea's tomography, a factor in the pathogenesis of keratoconus [4, 5].

Since first described by Luce [6] in 2005, the Ocular Response Analyzer (ORA, Reichert Ophthalmic Instruments, Depew, New York) has been widely used for* in vivo* assessment of corneal biomechanics [7–9]. Corneal hysteresis and the corneal resistance factor are the main biomechanical parameters measured by the Ocular Response Analyzer. Several studies [10–12] have compared the biomechanical properties of normal and keratoconic corneas and found that the latter have lower corneal hysteresis and resistance. However, these parameters are derived from a proprietary algorithm applied to the measured waveform, and the ORA cannot display the dynamics of the deformation process in real time. Thus, further research into technologies for measuring corneal stiffness and biomechanics is warranted.

Very recently, corneal visualization Scheimpflug technology (Corvis ST, Oculus, Wetzlar, Germany) has been developed to evaluate corneal biomechanics. This instrument displays corneal deformation in real time and records the deformation parameters for analyzing corneal biomechanics [13].

In the current study, we compared the corneal biomechanical parameters of keratoconus patients and normal controls using measurements obtained with the Corvis ST and estimated the sensitivity and specificity of these parameters for discriminating keratoconus corneas from normal corneas. To further evaluate the results obtained with the Corvis ST, we also applied Pentacam corneal tomography (Oculus, Wetzlar, Germany) to measure the anterior segment parameters.

## 2. Methods

### 2.1. Subject Recruitment

This prospective comparative study included 120 eyes of 107 participants: 60 keratoconic eyes from 47 keratoconus patients (the KC group) and 60 normal eyes from 60 controls (the control group). One randomly selected eye of each participant in the control group and one or two keratoconic eyes in the KC group were examined. A diagnosis of keratoconus was made if the eye had an irregular cornea determined by distorted keratometry mires or distortion of the retinoscopic or ophthalmoscopic red reflex and at least one of the following slit-lamp signs: Vogt's striae, Fleischer's ring with an arc >2 mm, or corneal scarring consistent with keratoconus [14–16]. Potential subjects were excluded from the study if they had undergone previous corneal or ocular surgery, had any ocular pathology other than keratoconus, or had systemic diseases known to affect the eye. Participants were instructed to remove soft contact lenses at least 72 hours and rigid contact lenses at least 1 month, before the examination. Data were collected from August 2012 to May 2013 at the Chinese General Hospital of the People's Liberation Army (PLA), Beijing, China. All participants signed an informed consent form in accordance with the tenets of the Declaration of Helsinki and this study received Institutional Review Board approval of Chinese PLA General Hospital, Beijing, China.

### 2.2. Ocular Examinations

All participants underwent a complete ophthalmic examination, including a detailed assessment of uncorrected distance visual acuity, corrected distance visual acuity, slit-lamp microscopy, and fundus examination, intraocular pressure using Goldmann applanation tonometry (IOP-GAT, Haag-Streit, Koenz, Switzerland), corneal topography (Allegro Topolyzer; Wavelight AG, Germany), corneal tomography (Pentacam), and corneal biomechanics (Corvis ST). All measurements were taken between 09:00 and 17:00 by 2 trained ophthalmologists during the same visit. Three effective results were obtained from each instrument and the mean was utilized for analyses.

### 2.3. Pentacam Measurement

The Pentacam system (software version 1.18r15) measured the corneal tomography using a rotating Scheimpflug camera as described preciously [17–21]. This camera captured 25 images of the anterior eye segment within 2 seconds by rotating 360 degrees around the optical axis of the eye in one measurement. Minute eye movements were captured by a second camera and were corrected simultaneously. A measurement with an “OK” quality-specification reading was accepted; otherwise the measurement was discarded and the examination was repeated.

The Pentacam output parameters were flat, steep, and mean keratometry; astigmatism; central corneal thicknesses; anterior chamber depth, volume, and angle; and corneal volumes at 3, 5, 7, and 10 mm (CV_3_ to CV_10_).

### 2.4. Corvis ST Measurement

**Figure 1:**
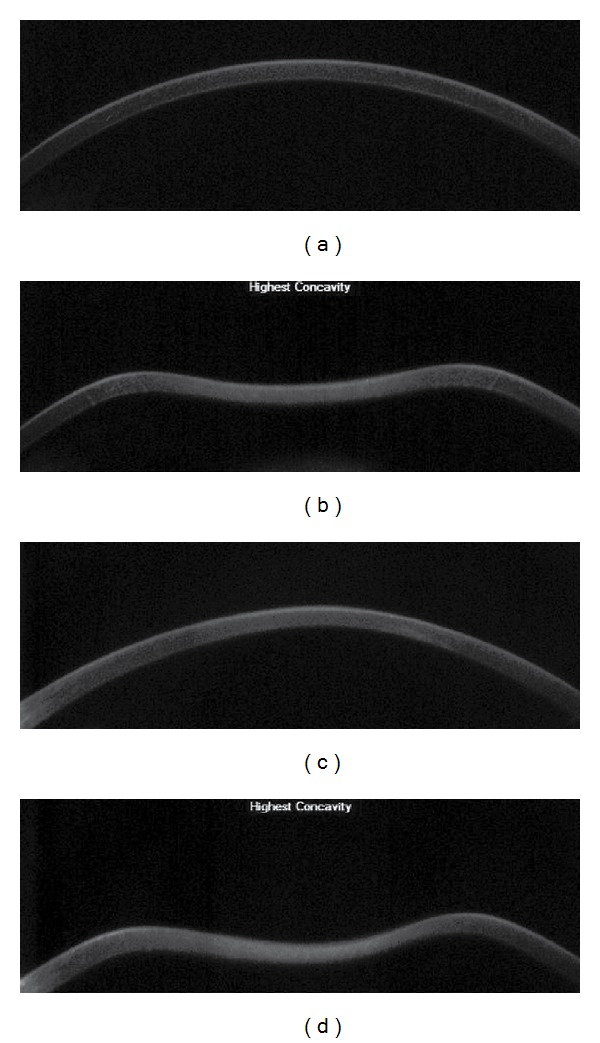
Cornea at stasis and maximum concavity in normal ((a) and (b)) and keratoconic ((c) and (d)) corneas. The convexity of the cornea at stasis and DA at maximum concavity is greater in the keratoconus than the normal corneas.

**Figure 2:**
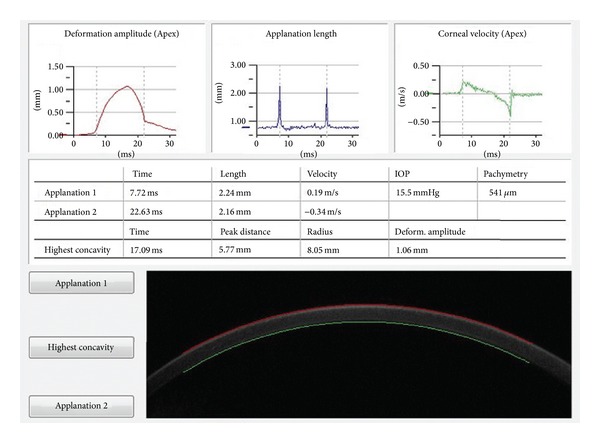
Corvis ST output. The output includes the IOP, CCT, and corneal biomechanical characteristics (applanation time, length, and velocity, time to the highest concavity and curvature radius, peak distance, and deformation amplitude).

The Corvis ST (software version 1.00r30) allows noninvasive imaging of the cornea's dynamic deformation response to a puff of air. A high-speed Scheimpflug camera records the deformation with full corneal cross-sections, which are then displayed in slow motion on a control panel (Figure 1); the camera records 4330 images/s and 8.5 mm horizontal coverage. The image resolution is as much as 640 × 480 pixels [22]. A representative output is shown in Figure 2, with several parameters related to the deformation process.

During the deformation response, a precisely metered air pulse causes the cornea to move inward or flatten (the phenomena of corneal applanation), that is, the first applanation. The cornea continues to move inward until reaching a point of the highest concavity. Because the cornea is viscoelastic, it rebounds from this concavity to another point of applanation (the second applanation) and then to its normal convex curvature. The Corvis ST records throughout the deformation process and therefore gains information concerning the cornea's viscoelastic properties and stiffness, as well as recording standard tonometry and pachymetry data [8]. Specifically, the Corvis ST outputs are IOP, central corneal thickness (CCT), time from the initiation of the air puff (time_0_) until the first applanation and second applanation (A-time_1_ and A-time_2_, resp.), length of the flattened cornea at the first applanation and second applanation (A-length_1_, A-length_2_), corneal velocity during the first and second applanation moments (*V*_in_, *V*_out_), time from the start until the highest concavity of the cornea is reached (highest concavity time), central curvature radius at the highest concavity (highest concavity curvature), distance of the two surrounding “knees” at the highest concavity (peak distance) as seen in cross-section, and maximum deformation amplitude (DA, from start to the highest concavity) at the corneal apex [23].

In the current study, we also used Goldmann applanation tonometry to measure the IOP and the Pentacam to detect the CCT, although the Corvis ST can measure both IOP and CCT.

### 2.5. Statistical Analysis

Statistical analyses were performed with SPSS version 17.0 software (SPSS for Windows, Chicago, IL). The Kolmogorov-Smirnov test was used to check for a normal distribution of quantitative data, which are here provided as the mean and standard deviation (SD). Differences between data were evaluated using Welch's modified Student's two-sample* t*-test and the Wilcoxon rank-sum test. A *P* value <0.05 was considered statistically significant. A receiver operating characteristic (ROC) curve was constructed to identify the overall predictive accuracy of biomechanical parameters and to calculate the sensitivity and specificity of these parameters. Pearson's correlation coefficient was used to evaluate the relatedness of the DA to corneal tomography parameters and the IOP-GAT.

## 3. Results

The mean age of patients in the KC group was 25.43 ± 6.05 years (range: 18 to 40 years) and in the normal control group was 26.6 ± 6.16 years (range: 19 to 42 years; *P* = 0.67, Wilcoxon rank-sum test). Most of the tomography and biomechanical characteristics of the keratoconic eyes were significantly different from those of the normal eyes (Table 1).

**Figure 3:**
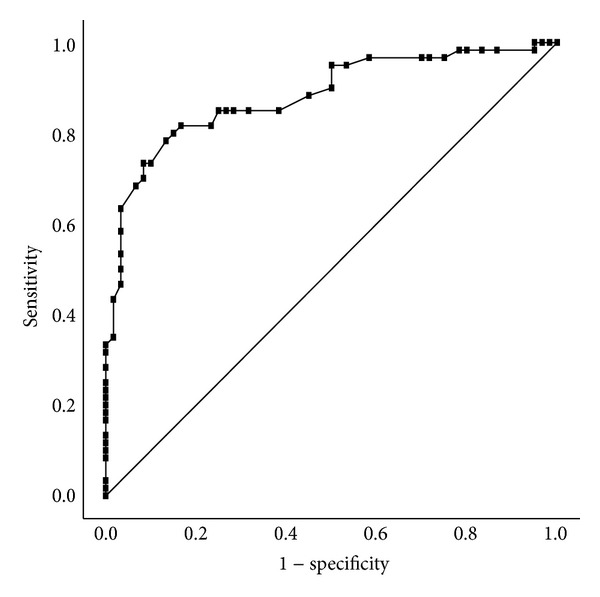
ROC curve (graphical plot of the sensitivity versus false positive rate) for DA. The cutoff was 1.18 mm, with 81.7% sensitivity and 83.3% specificity (test accuracy, 82.5%).

The ROC curve analysis showed that the DA had the greatest area under the ROC curve (AUC) among all biomechanical parameters for differentiating keratoconus from normal corneas. The AUC for the DA was 0.882 with an optimal cutoff point of 1.18 mm, sensitivity of 81.7%, specificity of 83.3%, and test accuracy of 82.5% (Figure 3).

**Figure 4:**
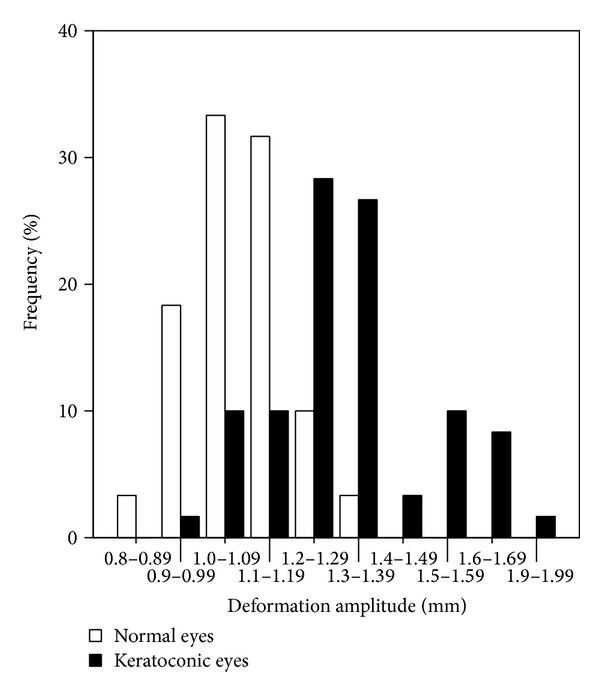
Histogram of DA for keratoconic and normal eyes.

The mean DA values were 1.32 ± 0.19 mm (range: 0.92 to 1.96 mm) in the KC group and 1.08 ± 0.11 mm (range: 0.87 to 1.33 mm) in the control group (*P* = 0, two-sample *t*-test; Figure 4). A shift of distribution of the DA to the right was observed for the KC group, although a significant overlap existed between 1.0 and 1.4 mm, indicating that the mean DA value in the KC group was higher than that of the control group.

**Figure 5:**
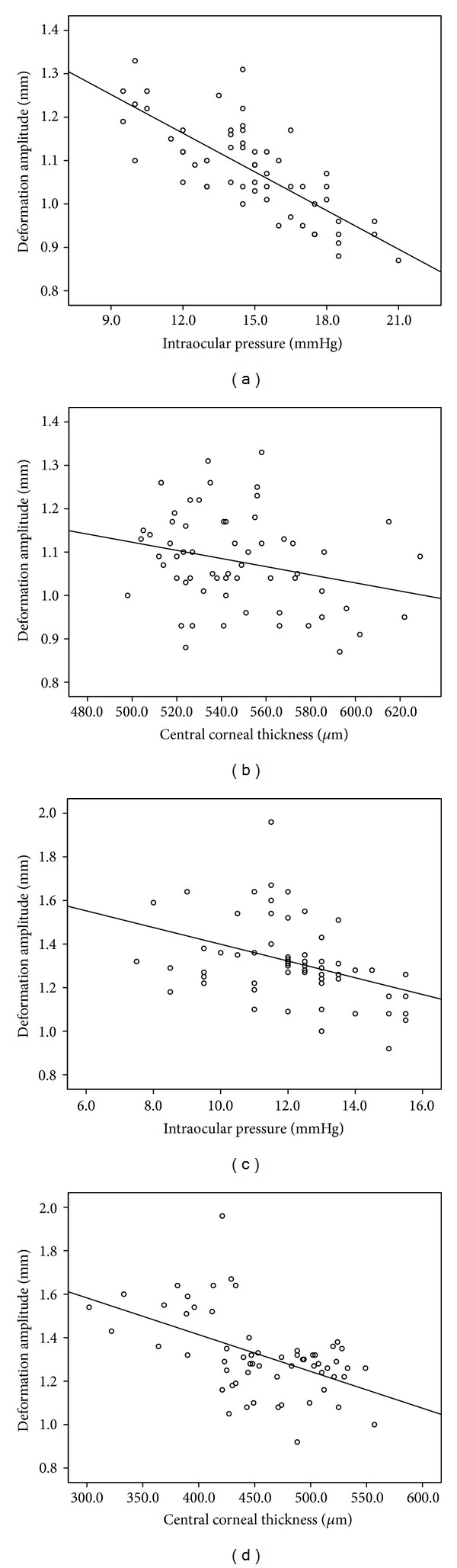
Scatterplots DA versus IOP-GAT (a) and CCT (b) in normal eyes and scatterplots of DA versus IOP-GAT (c) and CCT (d) in keratoconic eyes.

As shown in Table 2 and Figure 5, the DA negatively correlated with IOP-GAT, CCT, and CV at 3 and 5 mm in both groups (Table 2 and Figure 5).

## 4. Discussion

Keratoconus is an ectatic corneal disorder which can cause visual impairment by aggravating myopic and astigmatic conditions [1]. Keratoconus is considered a contraindication for most refractive surgeries, so accurate preoperative diagnosis is particularly important. However, it is sometimes difficult to diagnose keratoconus, especially forme fruste keratoconus, because of the lack of positive clinical signs. Usually, keratoconic eyes are discriminated from normal corneas using corneal topography and tomography [24, 25]; corneal biomechanical features are detectable before the manifestation of typical topographic signs [26].

The Corvis ST monitors the deformation process of the cornea in a cross-sectional view using an ultrahigh speed Scheimpflug camera, which makes it possible to visualize dynamic changes. Because the instrument is not yet widely used, the related clinical data are very limited. We conducted this study to compare the corneal tomography and biomechanical characteristics provided by the Pentacam and Corvis ST between patients with keratoconus and age-matched controls. We found that Corvis ST offers an alternative and viable method for measuring corneal biomechanical properties. The DA had the greatest AUC among all the biomechanical parameters, but with a significant overlap between the KC and control groups.

In this study, most of the tomography characteristics of keratoconus were significantly different from those of normal corneas. The keratometry values, astigmatism, anterior chamber depth, and anterior chamber volume were significantly higher in the KC group than in the control group, whereas the corneal thickness, anterior chamber angle, and corneal volume were lower in the KC group. As in some other studies, the corneal thickness and corneal volume were significantly lower in keratoconus patients than in normal cornea [15, 27].

The findings of our present study were consistent with those of Ambrosio et al. [23], specifically that the DA was significantly greater, concavity curvature was lower, and corneal applanation velocity was faster in the KC group than the control group. In addition, CCT and CV were less in the KC group than in the normal, which may result in less effective corneal collagen fibers in keratoconus. Given the major contribution of collagen fibers to corneal stiffness, this means that the corneal mechanical strength should be weakened in keratoconus, a concept that is supported by findings that showed less resistance to either air pulse indentation or IOP [28]. These may be reasons for the larger DA and lower concave curvature during the corneal indentation process and faster corneal velocity, during the two applanation moments in our study. The influence of the corneal thickness on the DA had also been demonstrated previously [29].

Although most of the biomechanical parameters were statistically different between the two groups, to differentiate keratoconus from normal corneas the DA was the most sensitive. Thus, we consider that the DA measured via Corvis ST is the most viable as a diagnostic parameter and deserves clinical attention. However, while the present study found that the DA is the best parameter for characterizing the biomechanical status of the cornea, the large overlap in ranges (1.0 to 1.4 mm) between the groups compromised its accuracy in discriminating keratoconus from normal corneas. Thus, the new biomechanical metrics should not be relied on as a stand-alone method for keratoconus diagnosis.

Our correlation analysis showed that the DA negatively correlated with IOP-GAT, CCT, and CV at 3 and 5 mm, in both groups. Hon and Lam [13] also found that the DA was negatively associated with CCT. Leung et al. [30] reported that a higher IOP and a greater CCT were associated with a smaller DA in 144 participants, with or without glaucoma. IOP is an important factor that affects the DA value, so, when using the new biomechanical parameter (i.e., DA) to display corneal characteristics, IOP needs to be taken into account carefully. Ambrosio et al. [23] reported that a combined parameter based on linear regression analysis (the Corvis Combo1) was better at distinguishing keratoconic eyes from normal eyes. The combined parameter is more representative of the effects of IOP on the deformation response and therefore more directly reflects the characteristics of the cornea itself.

Our study had some limitations. First, this was a cross-sectional study with measurements made only once. Second, the sample size was relatively small and hence statistical values may need to be interpreted with caution. Finally, the current Corvis ST software (the first generation with version 1.00r30) requires sophistication, and its outputs can only be regarded as raw data for characterizing corneas. To describe the biomechanical properties of the cornea, it is more useful to calculate the elastic modulus from the raw data. With advances in the understanding of biomechanics, the prospects for using the corneal deformation measurement will improve.

In summary, the corneal biomechanical metrics measured by the Corvis ST showed statistically significant differences between the keratoconus patients and normal controls. The DA was the most reliable indicator and may provide an additional reference for discriminating keratoconus from normal corneas. Additional research on this new technology is warranted to elucidate its full usefulness in clinical practice.

## Figures and Tables

**Table 1:** Comparison of tomography and biomechanical parameters between the KC and control group, mean ± SD (range).

	Control	KC	*P* value
Tomography			
Flat keratometry (diopters)	43.27 ± 1.56 (38.5–48.8)	48.47 ± 5.94 (40.1–69.3)	0^a^
Steep keratometry (diopters)	44.39 ± 1.61 (39.2–49.5)	52.09 ± 6.82 (40.8–72.9)	0^a^

Mean keratometry (diopters)	43.82 ± 1.54 (38.9–48.8)	50.18 ± 6.19 (40.8–71)	0^a^
Astigmatism (diopters)	1.12 ± 0.68 (0–3.7)	3.63 ± 2.72 (0.1–9.3)	0^a^

Central corneal thickness (*μ*m)	546.1 ± 30.09 (498–629)	456.37 ± 57.45 (302–557)	0^a^
Corneal volume at 3.0 mm (mm^3^)	3.94 ± 0.22 (3.6–4.6)	3.45 ± 0.33 (2.7–4.1)	0^a^

Corneal volume at 5.0 mm (mm^3^)	11.56 ± 0.64 (10.5–13.3)	10.57 ± 0.76 (9–12.4)	0^b^
Corneal volume at 7.0 mm (mm^3^)	24.89 ± 1.4 (22.7–28.7)	23.31 ± 1.54 (20.3–27)	0^b^

Corneal volume at 10 mm (mm^3^)	61.2 ± 3.67 (55.8–70.9)	58.18 ± 4.01 (50.6–68.3)	0^b^
Anterior chamber angle (degree)	39.14 ± 5.78 (28.4–63.6)	37.25 ± 5.71 (23.6–52.5)	0.074^b^

Anterior chamber depth (mm)	3.17 ± 0.32 (2.2–4.03)	3.43 ± 0.4 (2.16–4.39)	0^b^
Anterior chamber volume (mm^3^)	185.2 ± 36.73 (92–276)	203.18 ± 35.64 (124–263)	0.007^b^

Biomechanics			
A-time_1_ (ms)^c^	7.52 ± 0.43 (6.81–8.58)	7.04 ± 0.36 (5.91–7.74)	0^b^
A-length_1_ (mm)^d^	1.78 ± 0.27 (1.34–2.28)	1.69 ± 0.33 (0.98–2.35)	0.108^b^

*V*_in_ (m/s)^e^	0.15 ± 0.03 (0.08–0.24)	0.17 ± 0.04 (0.11–0.26)	0.026^a^
A-time_2_ (ms)^c^	22.18 ± 0.52 (21.27–23.3)	22.5 ± 0.55 (21.46–23.69)	0.001^b^

A-length_2_ (mm)^d^	1.9 ± 0.49 (1.01–2.86)	1.47 ± 0.46 (0.66–2.54)	0^b^
*V*_out_ (m/s)^e^	–0.39 ± 0.08 (–0.6 to 0.23)	–0.53 ± 0.15 (–0.88 to 0.24)	0^a^

Highest concavity time (ms)^f^	16.72 ± 0.49 (15.25–18.25)	16.67 ± 0.94 (11.32–17.79)	0.419^a^
Highest concavity curvature (mm)^g^	7.52 ± 1.05 (4.1–10.75)	5.59 ± 2.32 (2.75–16.83)	0^b^

Peak distance (mm)^h^	4.50 ± 1.43 (2.21–5.99)	4.50 ± 1.43 (2.15–6.29)	0.585^a^
Deformation amplitude (mm)^i^	1.08 ± 0.11 (0.87–1.33)	1.32 ± 0.19 (0.92–1.96)	0^b^

IOP-GAT (mmHg)^j^	14.85 ± 2.79 (9.5–21)	12.09 ± 1.92 (7.5–15.5)	0^b^

^a^Wilcoxon rank-sum test; ^b^independent two-sample *t*-test; ^c^A-time_1_ and A-time_2_: time from the start until the first applanation and second applanation, respectively; ^d^A-length_1_ and A-length_2_: length of the flattened cornea at the first applanation and second applanation; ^e^ *V*_in_ and *V*_out_: corneal velocities during the first and second applanation moments; ^f^ time from the start until the highest concavity of the cornea was reached; ^g^curvature radius of the highest concavity; ^h^distance of the two “knees” at the highest concavity; ^i^maximum deformation amplitude at the corneal apex, from start to the highest concavity; ^j^intraocular pressure measured by Goldmann applanation tonometry.

**Table 2:** Correlation coefficient between DA and tomography parameters and intraocular pressure.

	Control group		KC group	
	*r* value	*P* value	*r* value	*P* value
Flat keratometry	−0.03	0.818	0.450	0
Steep keratometry	−0.119	0.365	0.633	0

Mean keratometry	−0.074	0.573	0.545	0
Astigmatism	−0.232	0.075	0.608	0

Central corneal thickness	−0.263	0.042	−0.52	0

Corneal volume at 3.0 mm	−0.263	0.042	−0.431	0.001
Corneal volume at 5.0 mm	−0.262	0.043	−0.264	0.041

Corneal volume at 7.0 mm	−0.259	0.046	−0.065	0.624
Corneal volume at 10 mm	−0.258	0.046	0.059	0.654

Anterior chamber angle	0.166	0.206	−0.046	0.727
Anterior chamber depth	−0.003	0.983	0.211	0.105

Anterior chamber volume	0.009	0.943	0.030	0.823
IOP-GAT	−0.763	0	−0.395	0.002

IOP-GAT: intraocular pressure measured by Goldmann applanation tonometry.
